# Post-translational modifications of proliferating cell nuclear antigen: A key signal integrator for DNA damage response (Review)

**DOI:** 10.3892/ol.2014.1943

**Published:** 2014-03-05

**Authors:** QIONG ZHU, YUXIAO CHANG, JIN YANG, QUANFANG WEI

**Affiliations:** 1 Battalion Two of Cadet Brigade, Third Military Medical University, Chongqing 400038, P.R. China; 2 Department of Cell Biology, Third Military Medical University, Chongqing 400038, P.R. China

**Keywords:** proliferating cell nuclear antigen, ubiquitin, post-translational modification, small ubiquitin-related modifier, DNA damage response

## Abstract

Previous studies have shown that the post-translational modifications of proliferating cell nuclear antigen (PCNA) may be crucial in influencing the cellular choice between different pathways, such as the cell cycle checkpoint, DNA repair or apoptosis pathways, in order to maintain genomic stability. DNA damage leads to replication stress and the subsequent induction of PCNA modification by small ubiquitin (Ub)-related modifiers and Ub, which has been identified to affect multiple biological processes of genomic DNA. Thus far, much has been learned concerning the behavior of modified PCNA as a key signal integrator in response to DNA damage. In humans and yeast, modified PCNA activates DNA damage bypass via an error-prone or error-free pathway to prevent the breakage of DNA replication forks, which may potentially induce double-strand breaks and subsequent chromosomal rearrangements. However, the exact mechanisms by which these pathways work and by what means the modified PCNA is involved in these processes remain elusive. Thus, the improved understanding of PCNA modification and its implications for DNA damage response may provide us with more insight into the mechanisms by which human cells regulate aberrant recombination events, and cancer initiation and development. The present review focuses on the post-translational modifications of PCNA and its important functions in mediating mammalian cellular response to different types of DNA damage.

## 1. Introduction

Proliferating cell nuclear antigen (PCNA) is a member of the DNA sliding clamp family, which also includes the *Escherichia coli* DNA polymerase (pol) III β-subunit and phage T4 gene protein (1,2). As a DNA sliding clamp, PCNA with its high processivity is able to perform the essential tethering of the replicative DNA pol δ to the template DNA, which is required for the duplication of an entire genome. The crystal structure analysis of PCNA reveals a ring-shaped trimeric complex with marked six-fold symmetry, which encircles the double-stranded DNA that it freely slides along (3,4). This structural information provides a vital explanation for the stable association between PCNA and the cellular DNA without PCNA binding directly to it, as well as by what means the pol complex links to the DNA strand in a processive manner.

The proteins that interact with PCNA have been well mapped (5–7) and one of the most significant observations that has emerged regarding the investigation of PCNA-binding proteins is that a number of partners contain a conserved PCNA-binding motif, the PCNA-interacting protein (PIP)-box. As predicted, a number of PIPs are involved in various aspects of DNA replication and processing, possibly through the use of the sliding clamp properties of PCNA to mediate their interactions with DNA. Thus, the overlapping nature of the combining sites for these PCNA binding partners indicated that the various factors must act sequentially and coordinately to perform their functions (8). Subsequently, the conserved PCNA-binding motif may provide a regulatory mechanism to coordinate different aspects of DNA metabolism, such as the cell cycle checkpoints, DNA replication and repair.

Previous studies have revealed that PCNA is ubiquitinated in the response to several DNA damaging agents. This modification occurs at the Lys164 residue of PCNA (9,10) and it has been identified that modification of PCNA enhances the binding of translesion synthesis (TLS) pols, such as pol ι and pol η, enabling lesion bypass (11). Although ubiquitinated PCNA is considered to result in the recruitment of damage tolerance pols to the stalled replication forks, the definite mechanisms of this modification remain largely unknown. The present review focuses on the post-translational modifications of PCNA, and its cellular functions for DNA replication and repair. Specifically, the important role of modified PCNA in mediating mammalian cellular response to different types of DNA damage is highlighted.

## 2. Modification of PCNA by ubiquitin (Ub)

Ub is a highly conserved protein with a 76 amino acid residue polypeptide, and only three amino acid differences have been identified between the protein in yeast and human cells. The C-terminus of Ub contains a conserved glycine residue to enable attachment to substrates, which is activated in an ATP-dependent manner linked with the cysteine residue of the Ub-activating (E1) enzyme to form a thiol ester linkage (12). The activated Ub is transferred to the Ub-conjugating (Ubc) enzymes (E2) to form an additional thiol ester linkage. Subsequently, and dependent on the aid of a Ub ligase (E3), the Ub covalently attaches to the lysine residues of target proteins (13).

To the best of our knowledge, the ubiquitination of target proteins involves the concerted action of the E1, E2 and E3 enzymes. The E1 and E2 enzymes form thiol ester adducts with Ub and the two factors are essential for the ubiquitination of substrates. The majority of organisms possess a single E1, multiple E2 and even more E3 enzymes (14). Therefore, the substrates may be modified by a single Ub (termed monoubiquitination) or by multiple Ubs in the form of an isopeptide-linked polyUb chain. These various modifications, particularly mono- versus polyubiquitination, often lead to qualitatively different outcomes (15). Although the majority of known cases of Ub modification are focused on targeting substrate proteins to the proteasome for degradation, studies have shown that Ub-dependent proteolysis is crucial in the regulation of a number of other cellular processes, such as cell signaling, cell cycle progression and DNA repair. Therefore, variation in Ub signaling may result from the use of different lysine residues in polyUb chain assembly (16). For example, the Lys-48-linked chain signals target proteasomes for degradation, whereas the Lys-63-linked chain constitutes a non-degradative signal in various pathways, such as DNA damage response and ribosomal protein synthesis (17).

Previous studies have revealed that PCNA is a major target for Ub modification during DNA damage response signaling pathways. The modification of PCNA by a single Ub at the Lys164 residue, in response to DNA damage, is termed monoubiquitination, which is regulated by the RAD6–RAD18 E2–E3 complex (18). The E2 RAD6 collaborates with E3 RAD18 and attaches the Ub to substrate proteins. The RAD18 gene in *Saccharomyces cerevisiae (S. cerevisiae)* belongs to the RAD6 epistasis group, which is highly conserved throughout eukaryotes. RAD18 contains a middle zinc finger domain, a SAP domain and a RING-finger domain at its N-terminal. As a key factor in the postreplication repair (PRR) pathway, RAD18 forms a tight E2–E3 complex with RAD6 to promote PCNA ubiquitination, which in turn is a crucial cellular regulation mechanism of the PRR pathways that are conserved in eukaryotes (19–21). Previous studies have shown that monoubiquitination of PCNA at Lys164, catalyzed by the RAD6–RAD18 complex, signals for error-prone repair, possibly by promoting the recruitment of a TLS pol. Compared with the replicative polymerases, these TLS polymerases typically contain non-restrictive active sites and lack 3′-5′ proofreading exonuclease activity, which allows them to accommodate distortions in the DNA (18).

The monoubiquitination of PCNA at the conserved Lys164 by the RAD6–RAD18 complex has been reported in a wide variety of organisms. Polyubiquitination of PCNA may be observed in yeast and mammal cells, however, this modification is also located at the Lys164 residue. The same Ub modification serves as the substrate for the extension of a Lys63-linked polyUb chain, which requires a ternary complex composed of the RING E3 protein, RAD5, as well as the heterodimeric methyl methanesulfonate (Mms) 2-Ubc13 complex. Ubc13 is a canonical E2 enzyme, however, Mms2 belongs to a small family of E2 enzyme variant proteins, which resemble E2 enzymes but lack the defining E2 active site cysteine residue (15,17). The Mms2-Ubc13 complex functions as an E2 enzyme that is specialized for the assembly of Lys-63-linked polyubiquitination chains. Based on this genetic evidence, the modification of PCNA by Lys-63-linked polyUb chains is necessary for the induction of the error-free DNA repair pathway upon DNA damage response. The formation of the Lys63-polyUb chains upon the ubiquitination of PCNA at Lys163 protects cellular DNA against error-prone TLS-induced genomic mutations, presumably via a template-switching mechanism using the newly synthesized sister chromatid as a template to promote the recovery of the blocked replication forks (17,22). Notably, the same Lys164 residue of PCNA at which Ub modification occurs has also been identified to be a target for small Ub-related modifier (SUMO) modifications (23,24). Furthermore, the modifications by Ub and SUMO are induced by replication stress or DNA damage and promote the different branches of DNA damage bypass.

## 3. Modification of PCNA by SUMO

The SUMO protein shares ~18% of its amino acid sequence identity with Ub and the two proteins share a similar three-dimensional structure. The genetic features of SUMO proposed to influence the interactions of substrates with other proteins or DNA are considered to be antagonists of the Ub protein. The SUMO pathway is initiated by a SUMO-activating enzyme termed E1, which transfers the activated SUMO to the E2 conjugating enzyme, Ubc9. SUMO is subsequently transferred from Ubc9 to the substrate with the assistance of the E3 Siz1 pathway (25). Similar to Ub, SUMO conjugates with a lysine residue to target the substrate (26). However, unlike the target substrates in ubiquitination for degradation, sumoylation is involved in, and regulates, numerous cellular metabolism processes, such as transcriptional regulation, nuclear transport, apoptosis and protein stability (27).

In yeast, a reciprocal pull-down experiment using wild-type PCNA and mutant PCNA demonstrated that PCNA may be sumoylated at the Lys127 and Lys164 residues. In addition, the double mutant K127R and K164R of PCNA were demonstrated to disturb the sumoylation of PCNA below detectable levels (28). Lys127 is a hydrophobic residue, which has been postulated to be a SUMO modification consensus site and confirmed as a unique component of yeast PCNA (29). By contrast, the Lys164 residue of PCNA is the major modification site of SUMO and Ub, which is commonly observed between yeast and humans. The two residues are located on the outside rim of the trimeric PCNA ring and are positioned distally from the encircled DNA (30). Lys127 is located in a large loop of PCNA, which connects the two adjacent terminal domains of the PCNA monomer that subsequently mediate polymerase interaction with the connecting loop (31,32). This indicates that the SUMO modification of PCNA at this site may interfere with DNA polymerase binding to PCNA. Currently, it is hypothesized that the prominent binding site for SUMO protein conjugation is the Lys164 residue, which is conserved within yeast and mammals. A previous study demonstrated that when the Lys164 residue of PCNA is experimentally mutated the SUMO conjugation at the Lys127 residue is stimulated, indicating that modifications by PCNA and SUMO occur on the same molecule (33).

Biochemical investigations have demonstrated an association between ubiquitination and sumoylation (34). As the two modifiers compete for binding at the same lysine residue, studies have hypothesized that sumoylation at the Lys164 residue of PCNA may function as an antagonist for Ub proteins (35). However, additional studies have revealed that the sumoylation of PCNA at the Lys164 residue does not merely interfere with Ub modification, but appears to be involved in other functions with the substrates. As ubiquitination and sumoylation are reversible processes (26,36), a sequential regulation of ubiquitination and sumoylation for substrates may be predicted. Therefore, it appears reasonable to presume that the SUMO and Ub transforming mechanism may be a prevalent event for the cellular metabolism process.

Thus far, studies have provided evidence that PCNA monoubiquitination is required for error-prone TLS. PCNA K63-linked polyubiquitination governs the template switch-dependent replication of DNA lesions via an error-free pathway, whereas the modification of PCNA by SUMO prevents recombination and regulates the template switch (37). In *S. cerevisiae*, the helicase Srs2 is recruited via a conserved SUMO-interaction motif in the C-terminus of the Srs2 by sumoylated PCNA (38). The recruitment of DNA helicase Srs2 disrupts RAD51 single-stranded presynaptic filaments, thereby interfering with the homologous recombination (HR) (37,39). Furthermore, previous studies have shown that the expression of the Lys164 site in mutant PCNA leads to the increased formation of double-strand breaks (DSBs) in the RAD18(−/−) cell line where the effect of the RAD18-dependent Lys164 PCNA ubiquitination can be ruled out. In addition, the expression of the PCNA-SUMO fusion prevents DSB formation and inhibits recombination as a result of replication stalling at DNA lesions (40,41). These observations demonstrate the importance of the SUMO modification of human PCNA in preventing replication-fork collapse at DSBs and providing genome stability.

## 4. PCNA ubiquitination in PRR

PRR was first identified as a means for the repair of single-stranded gaps during the DNA replication process that is induced by ultraviolet (UV) light damage. In wild-type cells, PRR is accomplished by at least two downstream pathways with completely distinct biological outcomes. The first is the TLS pathway, which involves a number of non-classical DNA polymerases, such as the Y-family of DNA polymerases, which bypass specific DNA damage lesions using the unrepaired DNA strand as a template. The second pathway is proposed to involve a template switch mechanism, dependent on RAD5 and the Mms2-Ubc13 complex, which are considered to allow extension by transiently pairing the blocked nascent strand and the bypass of DNA damage via the recruitment of HR machinery (18,42). As TLS uses damaged DNA as a template and recruits low fidelity Y-family DNA polymerases (that frequently incorporate incorrect nucleotides during replication of the DNA damage site), the process is considered to be an error-prone pathway of DNA synthesis. By contrast, the template switch mechanism, which is considered to be a relatively error-free pathway, utilizes the newly synthesized sister chromatid as a template (43,44).

DNA synthesis by classical polymerases is frequently blocked by a variety of lesions, however, these replication blockages can be overcome by PRR pathways. In TLS, the stalled classical, replicative polymerase is replaced by a non-classical polymerase that is capable of replicating past the lesion. A previous study analyzing a yeast model clearly demonstrated the significant role of the PRR pathway in maintaining genomic stability (45). In addition, several lines of evidence have revealed that PCNA is pivotal for initiating and selecting the different bypass modes of PRR. In yeast, DNA damage, which is induced by the monoubiquitination of PCNA at the Lys164 residue, is mediated by the RAD6–RAD18 complex at a stalled DNA replication fork (9). PCNA monoubiquitination may also trigger the replacement of replicative polymerases with non-classical TLS polymerases, which are able to replicate past the DNA lesions. Furthermore, the Ub binding motif in the majority of TLS polymerases and/or the PCNA interaction motif appear to be significant in the regulation of the TLS pathway. The TLS pathway uses low fidelity DNA polymerases that usually repair the DNA in an error-prone manner since these polymerases have no proofreading activity. Several mammalian and yeast TLS polymerases have been completely identified, including pol η, REV1, REV3, pol ι and pol κ. These low fidelity polymerases allow replication past a variety of DNA lesions without repairing the damage (46). In addition, PCNA is further polyubiquitinated by the RAD5 and Ubc13-Mms2 pathways, which add a non-canonical Lys-63-linked polyUb chain onto the monoubiquitinated Lys164 residue of PCNA. Once modified by the polyUb chain, PCNA triggers TLS using a vague template switch mechanism, which involves the utilization of specific HR proteins and newly synthesized sister chromatids to bypass the DNA damage in an error-free manner. However, the synthesis achieved by these damage-tolerant polymerases remains controversial in higher eukaryotes (47). Furthermore, the sumoylation of PCNA at the Lys164 residue has been found to inhibit the template switch pathway. This antagonistic effect occurs as the sumoylated PCNA recruits a DNA helicase, termed Srs2 (23), which disrupts RAD51 nucleoprotein filaments that are fundamental to the initiation of HR.

Eukaryotes possess several low fidelity DNA polymerases, which differ from the classical polymerases in their ability to regulate damaged DNA templates. For example, the Y-family DNA pol η functions in the error-free TLS of the UV-induced formation of thymine dimers. By contrast, the DNA pol ζ functions in mutagenic TLS to bypass DNA lesions (48,49). At present, the best understood polymerase that is involved in TLS and tumorigenesis is pol η and the lack of pol η in humans results in a cancer-prone genetic disorder, the variant form of xeroderma pigmentosum. In mammal cells, pol η, Rev1, pol ι and ubiquitinated PCNA colocalize to the replication foci following DNA damage (50). In addition, in wild-type cells, pol η specifically interacts with the ubiquitinated PCNA following DNA damage, however, not with the unmodified PCNA. Thus, in the presence of ubiquitinated PCNA, the classical DNA pol δ on the DNA may be replaced by pol η when the replication fork stalls at the damaged DNA *in vivo* (51). The two branches of the PRR pathway, the error-free and the highly mutagenic branches, are likely to maintain a dynamic balance in cells. However, these branches are defective in the error-free PPR pathway of yeast cells and, therefore, spontaneous mutation rates may be elevated by 30-fold (52), which may be considered to be a cancer predisposition factor. Ubiquitinated PCNA mediates error-prone DNA synthesis, which has been postulated as a primary factor for genomic instability and cancer development, although, the direct evidence is minimal. Thus, PRR, which is a process that is orchestrated by ubiquitinated PCNA, appears to be critical for DNA damage tolerance.

## 5. PCNA ubiquitination in DSBs

DSBs are the most severe cytotoxic form of DNA damage, generated by ionizing radiation (IR), mechanical stress on chromosomes, radiomimetic chemicals, such as camptothecin (CPT), or the encounter of other types of DNA lesions by the replication machinery (53,54). As one of the most lethal forms of DNA damage, if repaired incorrectly or left unrepaired, DSBs result in chromosomal instability, which eventually leads to cell death or cancer genesis. DSBs are repaired by the HR pathway, which uses the newly synthesized sister chromatid as a template or by the non-homologous end-joining (NHEJ) pathway, which directly joins the broken DNA ends (55). Increasing evidence has implied that, in addition to its traditional functions to bypass DNA damage, ubiquitinated PCNA also functions in repairing DSBs in vertebrae. However, the exact manner in which ubiquitinated PCNA is involved in the DSB repair process remains unknown.

Previous *in vitro* and *in vivo* studies have revealed that PCNA ubiquitination may be activated as a result of multiple types of incidents. In mammal cells, studies have revealed that a wide range of DNA damage agents trigger PCNA ubiquitination, including alkylating agents (such as Mms), bulky adducts (for example, benzo(a)pyrene diol epoxide [BPDE]), crosslinking agents (such as cisplatin) and photoproducts induced by UV irritation (56–59). Previous studies have also indicated that modified PCNA observed at the blocked DNA replication forks or replication-independent events, such as DSBs induced by bleomycin and IR-induced DSBs that are not accompanied by base damage, do not trigger the ubiquitination of PCNA (60,61). Furthermore, in budding yeast, treatment with the topoisomerase inhibitor, CPT, which results in DNA replication fork stalling and even breakdown at the DNA damaged site, does not active PCNA ubiquitination (62). The results of a recent study showed that the modification of PCNA is clearly induced in budding and fission yeast following treatment with DSB mutagenic agents, IR or homothallic switching (HO) endonuclease. However, in mammalian cells treated with IR, PCNA ubiquitination was not detected. Therefore, further investigations are required to provide satisfactory explanations for these discrepancies (63). Although, data exists showing that DSBs induce PCNA ubiquitination, the different modes of DNA damage response mechanisms that are regulated by the PCNA ubiquitination pathway remain enigmatic.

Recent studies on budding yeast have reported that the use of agents to generate pure DSBs, such as HO endonuclease, induce the PCNA-REV1 interaction, which is mediated by the ubiquitinated PCNA. In addition, following the generation of pure DSBs induced by HO expression, the RAD6–RAD18 complex-mediated PCNA ubiquitination activates the Rev1- and pol ζ-dependent DSB repair pathways (64). The possible mechanism of this action may be due to a lack of NHEJ activity. As the simple rejoining of damaged DNA ends via the NHEJ pathway does not occur, the DSBs are processed by exonuclease activities to generate ssDNA tracts at the DSB ends. The new ssDNA tracts may generate gaps with 3′-termini upon which the PCNA is loaded by replication factor C (65). Once loaded, the PCNA is ubiquitinated by the RAD6–RAD18 pathway and in turn, the PCNA ubiquitination may stimulate the activities of nearby Rev1 or pol ζ. Thus, the RAD6–RAD18 and Rev1-pol ζ complexes accumulate at sites close to the DSB ends (64,66). PCNA ubiquitination may, therefore, provide a direct platform for the activation of TLS polymerases, pol ζ and Rev1, which are essential for the DSB repair pathway.

## 6. PCNA ubiquitination in the cell cycle checkpoint pathways

The cell cycle checkpoints are signal transduction pathways that respond to damaged DNA by inhibiting cell cycle progression (67). The cell cycle checkpoints also control the fidelity of eukaryotic cell division, by controlling the orderly progression of critical cell cycle events, such as DNA replication and chromosome segregation, as well as ensuring the proper repair of damaged DNA. The cell cycle delays, that are elicited by the checkpoint signaling pathways, enable the integration of cell cycle progression with DNA repair. Consequently, the cell cycle checkpoints are important for preserving the integrity of the genome.

Acting as one of the significant regulatory mechanisms responsible for sensing DNA replication stress and damage, the DNA replication-dependent S phase checkpoint is considered to be important for guarding the stabilization of stalled replication forks. The S phase checkpoint, known as a surveillance system that prevents the firing of late replication origins, controls chromosome replication, prevents cell-cycle progression to mitosis, and is important for detecting and responding to DNA damage and repair (68,69). Furthermore, as the modification of PCNA is significant for DNA replication to bypass DNA damage, the modification of PCNA is considered to be most relevant during the S phase cell cycle checkpoint. In addition, previous observations have indicated that PCNA in budding yeast, consistent with its replicative function in response to DNA damage, is modified primarily during the S phase, whereas DNA damage in the G1 or G2 phases does not generally trigger the ubiquitination of PCNA. This indicates that all modified PCNA predominantly arise from S phase cells, even in asynchronous populations (63).

Previous studies have provided evidence to demonstrate that ubiquitinated PCNA may be detected in haploid G1 cells treated with DNA interstrand cross-link (ICL) agents (70). The biochemical and genetic studies indicated that only monoubiquitinated PCNA is induced by ICL damage, with no detection of polyubiquitination or sumoylation. The likely explanation for this is that the blocked DNA synthesis, induced by ICL agents, leads to PCNA monoubiquitination, which may regulate the exchange of DNA pol δ to the error-prone pol ζ. In G1 cells, mutation of the conserved Lys164 of PCNA to arginine abrogates the capability of DNA pol ζ to associate with chromatin following ICL damage. However, the RAD5-Mms2-Ubc13 complex-mediated polyubiquitination of PCNA at Lys164 may lead to an alternative error-free template switch model following the generation of a sister chromatid that is likely to occur in the late S and G2 phases (71). In mammal cells modified with bulky adducts, for example by using BPDE, the S phase checkpoint pathway is elicited (72). In addition, when the DNA replication process encounters BPDE-induced bulky adducts during S phase, the covalent modification of PCNA actives the exchange of the replicative polymerases with damage-tolerant enzymes. Briefly, in yeast and mammalian systems, the RAD6–RAD18 complex mediates the S phase-dependent monoubiquitination of PCNA, which may lead to the regulated activation of DNA pol ζ in a DNA damage bypass-dependent manner (73).

## 7. Outlook

**Figure 1:**
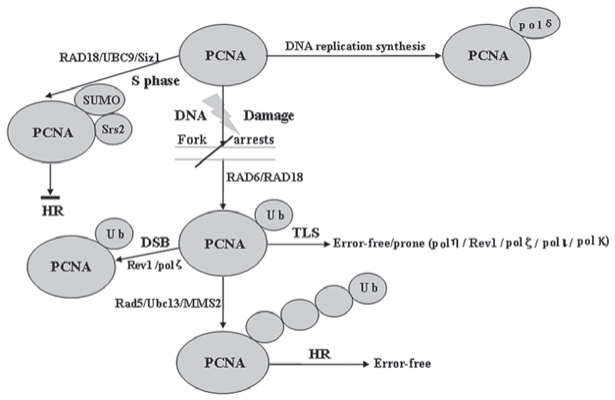
Role of Ub and SUMO modification of PCNA. PCNA may be modified by monoubiquitination, Lys-63-linked polyUb chains or SUMO at the same lysine 164 residue. PCNA monoubiquitination catalyzed by Rad6 and Rad18 directly activates TLS polymerases (such as pol η, Rev1 and pol ζ), which enable error-free or error-prone damage bypass, whereas Ubc13/Mms2 and Rad5 are required to extend the modification by a Lys-63-linked polyUb chain. PCNA polyubiquitination may occur if TLS fails, which subsequently results in a recombination-related error-free DNA damage tolerance pathway. Sumoylation of PCNA occurs in the S phase and attracts the antirecombinogenic helicase, Srs2, to inhibit unwanted recombination during DNA synthesis, however, ubiquitination of PCNA specifically occurs in cells with DNA damage or stalled replication. SUMO, small ubiquitin-related modifier; PCNA, proliferating cell nuclear antigen; pol, polymerase; TLS, translesion synthesis; UBC, ubiquitin-conjugating; DSB, double-strand break; Ub, ubiquitin; HR, homologous recombination.

A number of previous studies have analyzed the post-translational modifications of PCNA and revealed its importance in the DNA damage response and maintenance of genomic integrity. The modifications of PCNA are known to influence the choice of different pathways for the processing of DNA lesions during replication (Fig. 1). To the best of our knowledge, the monoubiquitination of PCNA at Lys164 by the RAD6–RAD18 complex may function to activate DNA damage tolerance pathways, whereas further extension of this modification mediated by RAD5 and the UBC13-MMS2 complex, termed polyubiquitination, triggers an alternative template switching mechanism (74–78). PCNA sumoylation also targets the same residue as that targeted in ubiquitination via the recruitment of Srs2 during the S phase, which serves to inhibit the HR pathways at the stalled replication fork.

In addition to its function as a sliding clamp that ensures the processivity of replicative DNA polymerases, PCNA serves as a binding platform for the various enzymes involved in DNA repair, chromatin assembly and cell cycle control (79). In the context of DNA replication and repair, SUMO and Ub jointly affect the key signal integrator, PCNA, at the replication fork. In response to DNA-damaging agents, PCNA is ubiquitinated at the highly conserved Lys164 residue (80). In *S. cerevisiae* yeast, the same lysine residue is modified by SUMO during the S phase, independent of any DNA damage. Therefore, the post-translational modifications of PCNA regulate the choice of the different modes of DNA bypass, depending on the species of ubiquitination, monoubiquitination of PCNA, activation of error-prone TLS and polyubiquitination, which may mediate an error-free template switching pathway (81). The present review discussed the possible regulatory mechanisms that control PCNA modifications, emphasizing the important role of modified PCNA during the replication of the DNA template onto which PCNA is loaded when activating the relevant Ub and SUMO conjugation factors. In addition, the review identified similarities, as well as significant variations among different organisms in the regulation of PCNA modifications.

In conclusion, despite the great advances that have been made in the understanding of PCNA ubiquitination in the DNA damage response pathways, a number of questions remain unanswered. These questions must be investigated in future studies to provide more detailed insights into the possible mechanisms by which PCNA ubiquitination and sumoylation function to regulate cell signal transduction pathways. In addition, further investigation may highlight the cellular coordination of these various modifications in the maintenance of cellular genomic integrity. A major challenge for the future, with regard to the integration of all these signals, is to develop a coherent model of the orchestration of the DNA damage response in time and space. An improved understanding of the effect of the mutual influences that the two relevant conjugation systems (ubiquitination and sumoylation) exert on each other is critically important to aid with the investigation of different post-translational modifiers, which are activated and utilized in a coordinated manner for the general preservation of genomic integrity.
